# Morphophysiological and transcriptome analysis reveal that reprogramming of metabolism, phytohormones and root development pathways governs the potassium (K^+^) deficiency response in two contrasting chickpea cultivars

**DOI:** 10.3389/fpls.2022.1054821

**Published:** 2023-01-11

**Authors:** Ankit Ankit, Ajeet Singh, Shailesh Kumar, Amarjeet Singh

**Affiliations:** National Institute of Plant Genome Research, New Delhi, India

**Keywords:** potassium, deficiency, chickpea, morphophysiology, transcriptome

## Abstract

Potassium (K^+^) is an essential macronutrient for plant growth and development. K^+^ deficiency hampers important plant processes, such as enzyme activation, protein synthesis, photosynthesis and stomata movement. Molecular mechanism of K^+^ deficiency tolerance has been partly understood in model plants Arabidopsis, but its knowledge in legume crop chickpea is missing. Here, morphophysiological analysis revealed that among five high yielding desi chickpea cultivars, PUSA362 shows stunted plant growth, reduced primary root growth and low K^+^ content under K^+^ deficiency. In contrast, PUSA372 had negligible effect on these parameters suggesting that PUSA362 is K^+^ deficiency sensitive and PUSA372 is a K^+^ deficiency tolerant chickpea cultivar. RNA-seq based transcriptome analysis under K^+^ deficiency revealed a total of 820 differential expressed genes (DEG’s) in PUSA362 and 682 DEGs in PUSA372. These DEGs belongs to different functional categories, such as plant metabolism, signal transduction components, transcription factors, ion/nutrient transporters, phytohormone biosynthesis and signalling, and root growth and development. RNA-seq expression of randomly selected 16 DEGs was validated by RT-qPCR. Out of 16 genes, 13 showed expression pattern similar to RNA-seq expression, that verified the RNA-seq expression data. Total 258 and 159 genes were exclusively up-regulated, and 386 and 347 genes were down-regulated, respectively in PUSA362 and PUSA372. 14 DEGs showed contrasting expression pattern as they were up-regulated in PUSA362 and down-regulated in PUSA372. These include somatic embryogenesis receptor-like kinase 1, thaumatin-like protein, ferric reduction oxidase 2 and transcription factor bHLH93. Nine genes which were down-regulated in PUSA362 found to be up-regulated in PUSA372, including glutathione S-transferase like, putative calmodulin-like 19, high affinity nitrate transporter 2.4 and ERF17-like protein. Some important carbohydrate metabolism related genes, like fructose-1,6-bisphosphatase and sucrose synthase, and root growth related Expansin gene were exclusively down-regulated, while an ethylene biosynthesis gene 1-aminocyclopropane-1-carboxylate oxidase 1 (ACO1) was up-regulated in PUSA362. Interplay of these and several other genes related to hormones (auxin, cytokinin, GA etc.), signal transduction components (like CBLs and CIPKs), ion transporters and transcription factors might underlie the contrasting response of two chickpea cultivars to K^+^ deficiency. In future, some of these key genes will be utilized in genetic engineering and breeding programs for developing chickpea cultivars with improved K^+^ use efficiency (KUE) and K^+^ deficiency tolerance traits.

## 1 Introduction

Among different nutrients required for optimum plant growth and development, potassium (K^+^) is an essential macronutrient. K^+^ is crucial for various plant physiological processes, including enzyme activation, photosynthesis, protein synthesis, stomata movement, carbon metabolism and starch synthesis (Maathuis, 2009; Wang and Wu, 2013; Luan et al., 2017). In addition, K^+^ plays an important role in maintaining turgor pressure and water homeostasis through K^+^ movement and vacuolar storage in plants (Maathuis, 2009). K^+^ status is also important for plants response to various abiotic and biotic stresses (Shabala and Pottosin, 2014; Nieves-Cordones et al., 2019; Deepika et al., 2022). Despite being the seventh most abundant element in earth crust, K^+^ availability for plants is very limited. Approximately, 98% of soil K^+^ exits in an unavailable state, such as primary (micas and feldspars) and secondary clay minerals, which cannot be absorbed and utilized by plants. Only 0.1-0.2% K^+^ exists as water soluble form that can be absorbed by plants through roots (Hafsi et al., 2014). Such low availability of K^+^ results in K^+^ deficiency which leads to several physiological and developmental changes in plants, such as change in root system architecture (RSA), chlorosis which further leads to necrosis at the tip and margins of older leaves, and overall hampered growth and yield (de Bang et al., 2021). In addition, Ribulose bisphosphate carboxylase activity is reduces, and photosynthesis process and stomatal conductance are hampered (de Bang et al., 2021). Globally, most arable field soils are K^+^ deficient for instance, about 75% of the paddy soils in China and 65% wheat fields in Southern Australia (Romheld and Kirby, 2010). In India, around 72% of the cultivated land soil is K^+^ deficient and requires immediate supply of K^+^ fertilizers to increase crop yield (Yadav and Sidhu, 2016). Unfortunately, due to low-K^+^ use efficiency, crop plants utilize only about 50% of applied K^+^ fertilizer (Britto and Kronzucker, 2008). Most of the unutilized fertilizer accumulate in the soil and a portion of it leaches down into the water bodies which adds to soil and water pollution, and also contributes in eutrophication (Nasr Esfahani et al., 2021). Collectively, high cost of K^+^ fertilizers and their bad impact on environment and human health urge for developing crop plants with high K^+^ uptake (KUpE) and use efficiency (KUE).

Chickpea (*Cicer arietinum* L.) is one of the most important legume crops known for its high nutritional value (Sagar et al., 2021). It is a self-pollinated diploid (2n = 2x = 16) cool seasoned annual pulse crop with a 738 Mb genome (Varshney et al., 2013). Among pulses, chickpea ranks third with the global production of around ~11.6 million tons *per annum*, out of which 80% is desi chickpea and 20% is Kabuli (Merga and Haji, 2019). Chickpea grain is very popular around the globe as nutrient rich seeds containing carbohydrates (50–58%), protein (15–22%), moisture (7–8%), fat (3.8–10.20%), micronutrients (<1%), amino acids like lysine and arginine, and a range of isoflavones (Jukanti et al., 2012). Importantly, India alone contributes about 70% of world’s chickpea production (FAOSTAT, 2016). However, as mentioned earlier, a major proportion of soil in Indian cultivated land is deficient in K^+^. Growing chickpea in K^+^ deficient soil will negatively affect plant growth and development. In addition, K^+^ deficiency in soil will make the plants prone to biotic and abiotic stresses (Shi et al., 2018; Nieves-Cordones et al., 2019). Collectively, these adverse effects of K^+^ deficiency may severely hamper chickpea production and worldwide chickpea supply. Unfortunately, the knowledge of molecular mechanism of K^+^ deficiency tolerance and K^+^ uptake and homeostasis related genes in chickpea is missing. To develop improved chickpea cultivars with better KUE and K^+^ deficiency tolerance, it is important to identify key K^+^ uptake and homeostasis related genes. Thus, we decided to undertake this study to understand molecular mechanism of K^+^ deficiency tolerance in chickpea. The major objectives of this study are first, to analyse the response of different chickpea cultivars to K^+^ deficiency and discover K^+^ deficiency sensitive and tolerant cultivars. Second, to identify the key K^+^ deficiency responsive genes and signaling pathways in contrasting chickpea cultivars to comprehend the molecular mechanism of K^+^ deficiency response and tolerance in chickpea.

Here, we performed the morphophysiological analysis of five high yielding desi chickpea cultivars (PUSA256, PUSA362, PUSA372, ICC1882 and ICC4958) under K^+^ sufficient and deficient conditions. Based on their response to K^+^ deficiency, PUSA362 was identified as K^+^ deficiency sensitive and PUSA372 as K^+^ deficiency tolerant cultivar. To understand the molecular basis of their contrasting response to K^+^ deficiency, RNA-Seq based comparative transcriptome analysis was performed in these two chickpea cultivars. K^+^ deficiency responsive DEGs were identified and categorized into different functional categories based on annotations. RNA-Seq expression data was validated for few selected genes using RT-qPCR analysis. KEGG pathway analysis was performed to identify different signaling pathways affected due to K^+^ deficiency in both the cultivars. Overall, this study will help to comprehend the molecular mechanism of K^+^ deficiency tolerance in important legume crop chickpea.

## 2 Materials and methods

### 2.1 Plant material and growth conditions

Seeds of desi chickpea (PUSA256, PUSA362, PUSA372, ICC1882 and ICC4958) were surface sterilized and grown as described by Deepika et al. (2022). Briefly, seeds were dipped in 70% ethanol for a minute followed by two times stringent wash with sterile water. This was followed by washing with 2% sodium hypochlorite solution containing two drops of tween-20 for 15 min. Seeds were further washed many times with sterile water and left overnight in sterile water. Seeds were then kept for germination on wet Whatmann filter for 2 days in dark. Uniformly germinated seeds were transferred to 1/4^th^ strength Hoagland media containing macronutrients- NH_4_H_2_PO_4_ (250μM), KNO_3_ (1.5 mM), Ca(NO_3_)_2_.4H_2_O (1mM), MgSO_4_.7H_2_O (0.5 mM), micronutrients- H_3_BO_3_ (10.6 μM), ZnSO_4_.7H_2_O (0.19 μM), CuSO_4_.5H_2_O (0.08 μM), H_2_MoO_4_·H_2_O (0.03 μM), MnCl_2_.4H_2_O (2.29 μM) and Na-Fe-EDTA (3.41 μM), at pH 5.5. For K^+^ deficiency, germinated seeds were transferred to media with 10 μM K^+^ (KNO_3_) and for K^+^ sufficiency, 1.5 mM K^+^ (KNO_3_) and grown for ten-days. Growth media (with sufficient K^+^ and deficient K^+^) was replenished after every two days. Growth conditions were maintained at 12/12 h photoperiod, temperature- 23°C/18°C, 200–300 μM photons/m^2^/s photon density and ~70% relative humidity. Morphological data from three replicates were captured where each replicates represent ten uniformly grown seedlings. Growth parameters, such as primary root length and shoot length were also measured from these seedlings. Based on morphological analysis, two chickpea cultivars (PUSA362 and PUSA372) were selected for further experiments.

### 2.2 K^+^ content measurement using ICP/MS

Uniformly germinated seeds of chickpea (PUSA362 and PUSA372) were grown for seven-days in 1/4^th^ Hoagland media. Then, half of the seedlings were transfer to K^+^ sufficient (1.5 mM) and half to K^+^ deficient (10μM) media. Samples (root and shoot separately) of both chickpea varieties were collected after ten-days of growth. Each sample was lyophilized and powdered, and 50mg powdered sample was dissolve in 8ml concentrated nitric acid (HNO_3_) in a separate digestion tube. The samples were digested in an oven at 180°C for 35 min followed by cooling. After this, volume of each sample was made up to 50ml with sterile water. The samples were then diluted in 1:9 ratio (v/v) using 2% HNO_3_. Ion content analysis in each sample was performed in ICP-MS machine (Agilent 7800). Three biological replicates for each root and shoot sample were used for the measurement of K^+^ content.

### 2.3 RNA isolation and processing

Ten- days old chickpea seedlings grown in K^+^-sufficient and K^+^-deficient conditions were harvested and immediately frozen in liquid nitrogen, and stored at -80°C till RNA isolation. Total RNA was extracted from three biological replicates of each sample according to Sagar et al. (2020). RNA samples were treated with DNaseI (Thermo-Scientific) to remove any genomic DNA contamination, and samples were further purified using RNeasy MinElute Clean-up Kit (QIAGEN). Quantification of RNA samples was done using nano-spectrophotometer (Nano Drop ONE^c^ -Thermo Scientific) and RNA integrity was checked by loading the RNA samples on 1.2% denaturing gel in 1X MOPS buffer. RNA quality was further ensured using an Agilent 2100 RNA Bioanalyzer (Agilent, USA) and samples with RNA integrity number (RIN) > 8 were used for RNA-Seq analysis.

### 2.4 Library preparation for RNA-Seq and data pre-processing

The library was prepared for RNA-seq experiment using the NEB Next Ultra II RNA Library Prep Kit (NEB, Massachusetts, USA) using manufacturer instructions. Three biological replicates of each sample (K^+^ sufficient and K^+^ deficient) were used for RNA-seq analysis. The prepared library was quantified using Qubit 3.0 fluorometer (Thermofisher Scientific, Massachusetts, USA) using DNA HS assay kit (Thermofisher Scientific, Massachusetts, USA). The insert size of the library was assessed using 4200 TapeStation (Agilent Technologies, CA, USA). Prepared RNA library was sequenced in Illumina Novaseq 6000 at Nucleome Informatics, Hyderabad, India. The raw RNA-seq reads in FASTQ format from all samples were subjected to quality filtering by using fastp v0.21.0 for the removal of low-quality reads (Phred score < 15; N base limit > 5) and reads with adapter contamination. The RNA-seq data has been submitted in NCBI with accession No.- PRJNA883591.

### 2.5 Differential gene expression analysis and functional annotation

The filtered RNA-seq data were analyzed using the new Tuxedo, an open-source pipeline (Pertea et al., 2016). The chickpea genome index was built by ‘hisat2-build’ utility of Hisat2 (v2.2.1) (Kim et al., 2019) by using chickpea genome (ASM33114v1), and splice and exon sites were extracted from genome annotation file. The filtered reads from all the samples were aligned to the indexes by using Hisat2 at default parameters. Further, the Stringtie (v2.1.5) software package (Pertea et al., 2015) was used for the transcriptome assembly, and quantification of expressed genes and transcripts. For each sample, the transcripts and their isoforms were assembled in separate GTF format files by using sorted BAM file as input. The transcript structures in all samples of each chickpea cultivars (PUSA362, and PUSA372) were merged by using StringTie. The merged GTF file for each sample was used for the re-estimation of transcript abundance. Gene count matrix was designed by a package Isoform Switch Analyze R (v1.18.0) (Vitting-Seerup and Sandelin, 2019) by using transcript sequences and counts data for each sample. The differential gene expression analysis was performed by using DESeq2 (v1.30.1), which normalizes libraries based on the geometric mean of the read counts, and then calculates the log2-fold change between defined conditions of samples. Differentially expressed genes (DEGs) with a log_2_-fold change ±1 with p-value < 0.05 were considered significant. For functional annotation of genes, the nucleotide sequence was used as input in the functional annotation pipeline of Blast2Go software package, performing the blast, mapping, and annotation of query sequences on various selected databases.

### 2.6 KEGG pathway analysis

Kyoto Encyclopedia of Genes and Genomes (KEGG) pathways were identified for DEGs having significant p-values. KEGG Mapper – Convert ID tool (https://www.genome.jp/kegg/mapper/convert_id.html) was used to convert NCBI gene ID (accession numbers) of each DEG to KEGG identifiers (cam ID). These KEGG identifiers were mapped to the known pathways using KEGG Mapper search tool (https://www.genome.jp/kegg/mapper/search.html). The search mode was selected as “cam”, which is the KEGG organism code for *Cicer arietinum*.

### 2.7 RT- qPCR analysis

To validate the RNA-seq expression data, RT-qPCR analysis was performed for few selected genes. RNA samples used for RNA-seq (K^+^ sufficient and K^+^ deficient conditions) were also used for cDNA synthesis to perform RT-qPCR. 1µg RNA was used in a 20µl reaction for the preparation of single-strand cDNA using iScript™ cDNA Synthesis Kit (Bio-Rad) as per the manufacturer’s instructions. Primers for selected DEGs were design in PRIMER EXPRESS software (PE Applied Biosystems, USA). Details of all primers are given in **Table S1**. The specificity of primers was confirmed by BLAST tool on NCBI and through dissociation curve analyses post RT-qPCR, as per protocol given in Singh and Pandey (2015). The expression of DEGs was detected using iTaq Universal SYBR Green super mix (Bio-Rad) according to the manufacturer’s protocol in Bio-rad CFX96 Real-time PCR machine (Bio-Rad). Three biological replicates of each sample, and three technical replicates for each biological replicate were used for expression analysis. *EF1α* gene was used as endogenous control for normalization of variance among samples. Expression data was analyzed through ΔΔCt method and the average fold change was plotted on the bar graphs with the standard error.

### 2.8 Statistical analysis

For statistical significance of the data, all experiments including morphophysiological, expression and quantitative analysis were performed in triplicates. Mean of the morphological observations, K^+^ content and expression values ± S.D (standard deviation) are represented in graphs. A two tailed student’s t-test was performed for statistical significance among three replicates. The data were considered statistically significant when p-value <0.05 (denoted by *), p-value <0.01 (denoted by **) and p-value <0.005 (denoted by ***). In transcriptome analysis, DESeq2 (v1.30.1) R package was used to normalizes libraries based on the geometric mean of the read counts. DEGs with Log_2_-fold changes of ±1 and p-value < 0.05 were considered statistically significant.

## 3 Results & discussion

### 3.1 Morphophysiological analysis of chickpea cultivars under K^+^ deficiency

**Figure 1:**
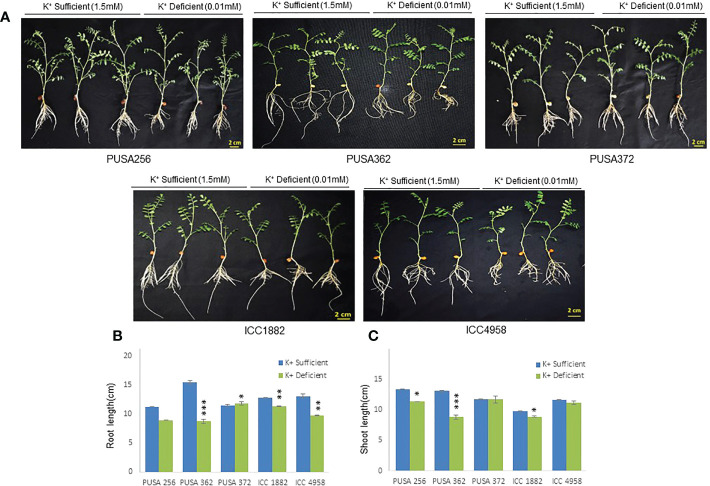
Phenotype analysis of five high yielding desi chickpea cultivars under K^+^ deficiency. **(A)** Germinated seeds of desi chickpea (PUSA256, PUSA362, PUSA372, ICC1882 and ICC4958) were transferred to growth media with K^+^ sufficient (1.5mM K^+^) and K^+^ deficient (0.01mM K^+^) conditions for ten days. Phenotype was recorded after ten days of growth. **(B)** Root and **(C)** Shoot length of five chickpea cultivars under K^+^ sufficient and K^+^ deficient growth conditions. X-axis shows the name of chickpea cultivar and Y-axis shows the root/shoot length (cm). Each analysis was repeated three times with 10 seedlings in each sample. Asterisk (*) indicates the p-value < 0.05, and (**) p-value < 0.01 and (***) p-value < 0.005 calculated using student t-test to determine statistical significance among samples.

**Figure 2:**
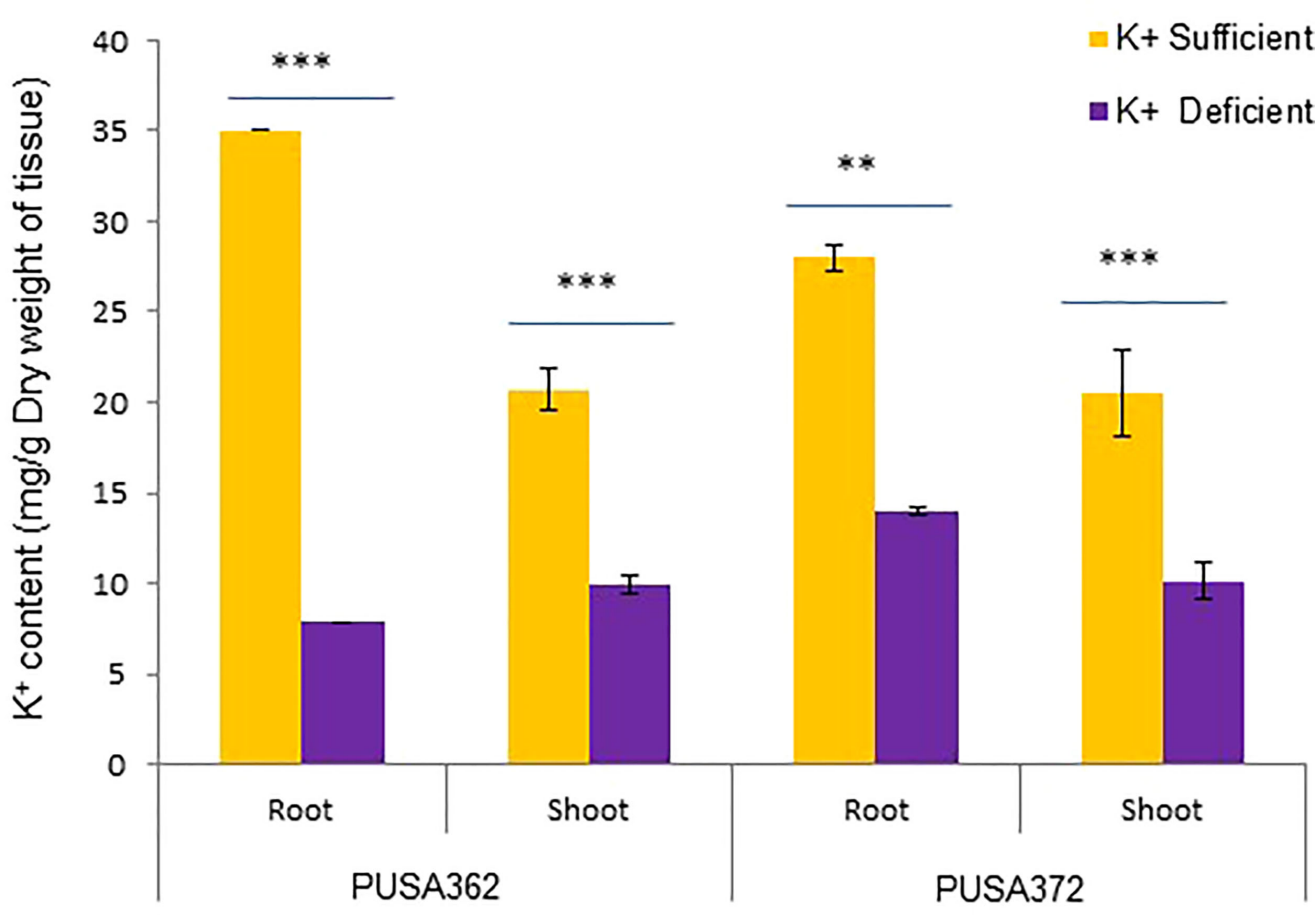
Total K^+^ content in PUSA362 and PUSA372 determined using ICP-MS analysis. Graphs showing the measurement and comparison of the K^+^ ion content of root and shoot samples in desi chickpea cultivars, PUSA362 and PUSA372 in response to control (1.5mM K^+^) and K^+^ deficient (0.01mM K^+^) condition. Each bar depicts the average of K^+^ content from three biological replicates of each sample. Standard error shown on bars was calculated from the standard deviation among the biological replicates. Asterisk (**) indicates the p-value < 0.01, and (***) p-value < 0.005 calculated using student t-test to determine statistical significance among sample.

The genetically diverse germplasm is essential for breeding and development of new cultivars with desirable traits such as KUE and K^+^ deficiency tolerance. Identification of genotypes with K^+^ deficiency tolerant traits has provided the suitable donor parents in several crop species (Tian et al., 2008; Wu et al., 2011). In this study, we performed the morphological analysis to evaluate and compare the effect of K^+^ deficiency on five high yielding improved desi chickpea cultivars (PUSA256, PUSA362, PUSA372, ICC1882 and ICC4958). The visible symptoms of long-term K^+^ deficiency include chlorosis at the tip of oldest leaves which turns into marginal necrosis (Marschner, 1995). However, mild or short duration K^+^ deficiency in crop plants does not leads to visible symptoms immediately, possibly due to the significant redistribution of nutrients between mature and young developing tissues. Initially, only a reduction in growth rate is observed and, later chlorosis and necrosis begin to appear in mature leaves (Mengel et al., 2001). Accordingly, numerous studies have analyzed the root growth pattern as an effect of K^+^ deficiency. It has been shown that K^+^ deficiency leads to shortened primary and lateral root length along with reduction in overall growth in plants, such as Arabidopsis, rice, foxtail millet, sweet potato, pears (*pyrus betulaefolia*) and tomato (Singh et al., 2018; Zhao et al., 2018; Cao et al., 2019; Ragel et al., 2019; Yang et al., 2020). Thus, here we suitably analyze the symptoms of early K^+^ deficiency i.e., change in plant growth rate, especially root and shoot growth. Out of five cultivars, PUSA362 seedlings were found to have shortest primary and lateral root length, reduced root hair numbers and stunted plant growth under K^+^ deficiency when compared to sufficient K^+^ condition (**Figure 1A**). The average height of PUSA362 plants in K^+^ sufficient conditions was about 28.5cm whereas, it was approx. 17.6cm in K^+^ deficient conditions. The average primary root length of PUSA362 was 15.5 cm in K^+^ sufficient conditions whereas, under K^+^ deficiency the average root length was found to be 8.8 cm (**Figure 1B** and **Table S2**). Similarly, the average shoot length was 13.0 cm in K^+^ sufficient conditions whereas, it was only 8.5 cm under K^+^ deficiency (**Figure 1C**). Among all the chickpea cultivars, K^+^ deficiency had the negligible effect on PUSA372 as it showed no major changes in root and shoot growth under K^+^ deficient and sufficient conditions. Its average primary root length was 11.53 cm and 11.9 cm, respectively under K^+^ sufficiency and deficiency (**Figure 1B**), and shoot length was 12.0 cm and 11.7 cm under K^+^ sufficiency and deficiency (**Figure 1C**). Further, ICP/MS analysis for K^+^ content measurement in root and shoot of both cultivars provides insights into the K^+^ translocation and storage status of chickpea plants under K^+^ deficiency. Under sufficient K^+^ conditions, the K^+^ content in the root of PUSA362 was 35mg/g of dry weight, while under K^+^ deficiency it was found to be 7.8mg/g (**Figure 2**). It infers that there is about 78% lesser K^+^ content in roots of PUSA362 under K^+^ deficiency. On the other hand, the K^+^ content in the root of PUSA372 under K^+^ sufficient and K^+^ deficient conditions was 27.9mg/g and 13.9 mg/g, respectively, indicating 50% lesser K^+^ under K^+^ deficiency. Therefore, reduction in root K^+^ content was more significant in PUSA362 than in PUSA372. This indicates that PUSA362 cultivar is less efficient in K^+^ uptake and storage in the root. In contrast, PUSA372 could uptake and store higher amount of K^+^ in the roots. Interestingly, shoot in both the cultivars had similar K^+^ content under both, K^+^ sufficient (~20mg/g of dry weight) and deficient conditions (~10mg/g of dry weight). It is observed that under K deficiency, alongwith shortened root length, the root endodermis becomes suberized. The suberization process is triggered by ABA (Barberon et al., 2016) and it interferes in K^+^ translocation (Chen et al., 2019). This possibly accounts for the significantly reduced K^+^ content in root and shoot of PUSA362 under K^+^ deficiency. Also, K^+^ has a stimulatory effect on the plasma membrane located ATPase in the sieve tube. The proton pumping ATPase generates a transmembrane potential gradient and pH gradient between the lumen of the sieve tube. This gradient effectively drives the transport of sucrose from the apoplasm into the sieve tubes. Thus, K^+^ plays an important role in phloem loading and phloem transport (Marschner, 1995; Römheld and Kirkby, 2010). The phloem sap which is transported from the mature leaf (source) to sink sites (roots) contains K^+^ along-with Mg^2+^, amino acids and sucrose as its major constituents (Jeschke et al., 1997). Sufficient amount of K^+^ in the leaves is thus, crucial for supplying sucrose to the roots for investing in energy expenditure for root growth and development (Cakmak et al., 1994; Omondi et al., 2020). In this study, relatively low shoot K^+^ content in PUSA362 may have hampered the phloem transport and sucrose supply to the roots and resulting in reduced root growth. Overall, the morphophysiological analysis concluded that PUSA362 is a K^+^ deficiency sensitive chickpea cultivar while, PUSA372 is K^+^ deficiency tolerant. Due to their contrasting response to K^+^ deficiency, these two chickpea cultivars were selected for global transcriptome analysis to understand molecular mechanism of K^+^ deficiency response in chickpea.

### 3.2 RNA-seq analysis reveals K^+^ deficiency related transcriptome in chickpea cultivars

**Figure 3:**
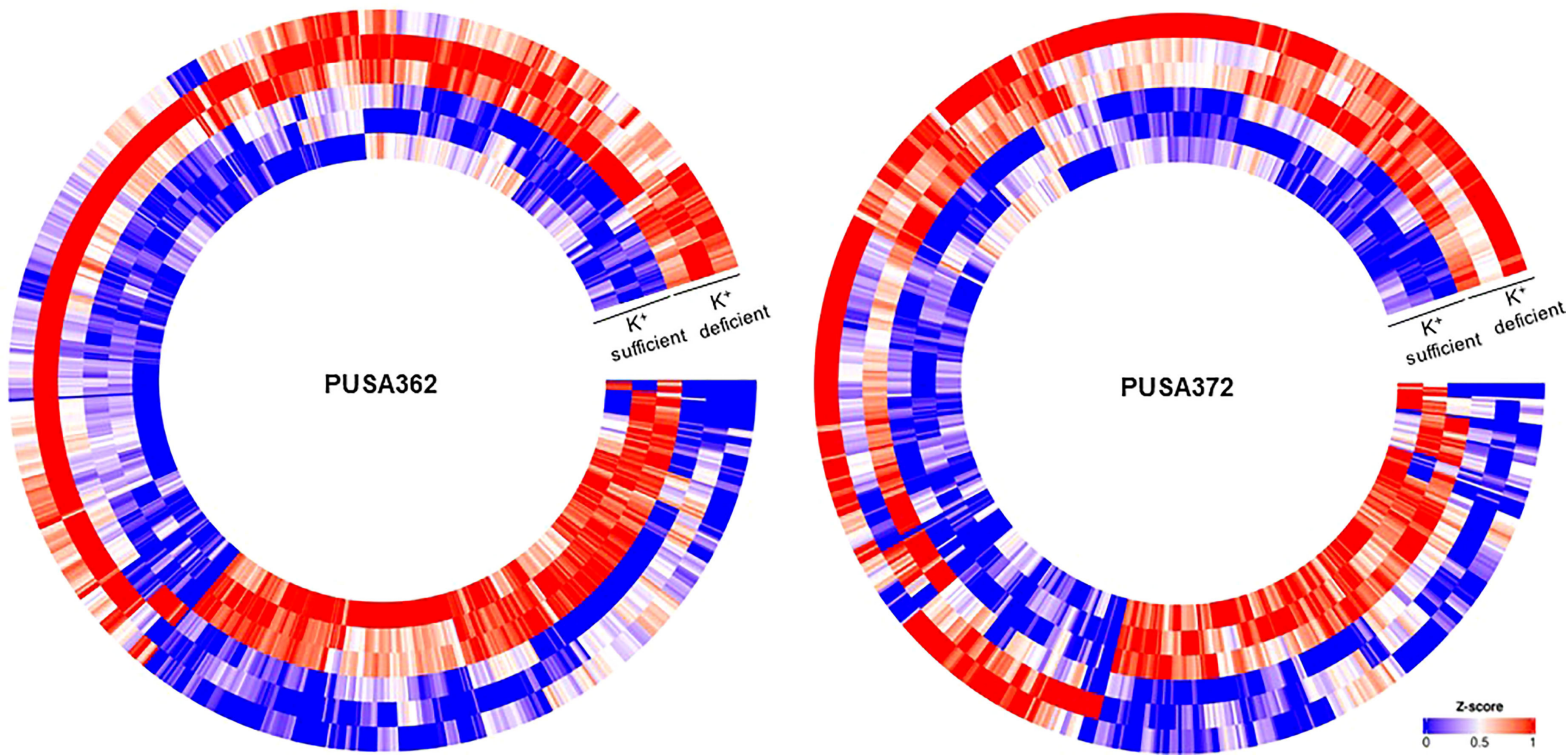
Heat map depicting complete set of DEGs of PUSA362 and PUSA372 under K^+^ deficiency. Each heat-map is specific for chickpea cultivars PUSA362 and PUSA372. Three circles each of K^+^ sufficient and K^+^ deficient conditions represent three replicate biological samples of these conditions. Heat-map is scaled with Z-score (0-1) indicating the expression of genes across the samples.

**Figure 4:**
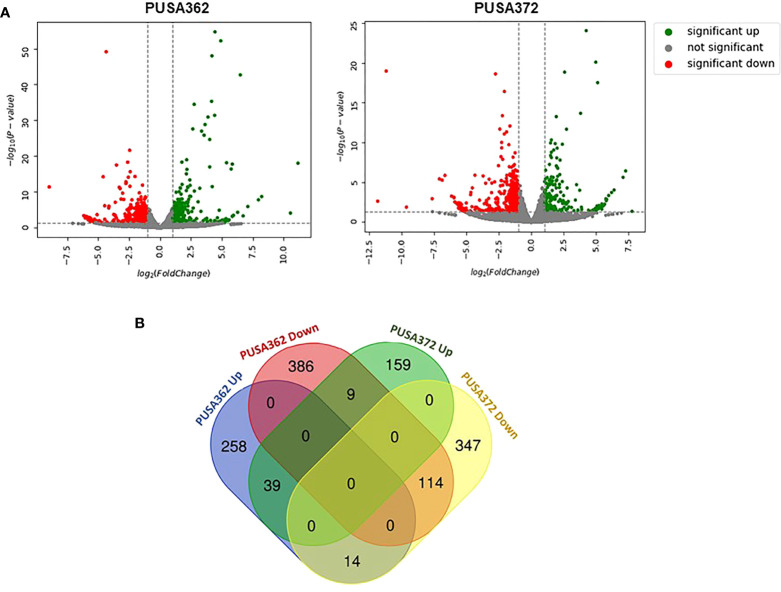
Summary of specific and overlapping expression pattern of DEGs in PUSA362 and PUSA372. **(A)** Volcano plots provides the summary of expression regulation of DEGs in PUSA362 and PUSA372 in response to K^+^ deficient condition. Green dots represent significantly up-regulated DEGs, red dots represent significantly down-regulated DEGs and grey dots refers to all genes which are not significantly expressed. X-axis shows the log_2_fold change of gene expression and Y-axis indicates the statistical significance in terms of -log_10_(P-value). **(B)** Venn diagram showing the number of unique as well as commonly up-regulated and down-regulated DEGs in PUSA362 and PUSA372 variety in response to K^+^ deficient condition. Mentioned numbers in each block indicates the total DEGs in corresponding group.

Global transcriptome analysis is one of the popular methods to identify the key genes associated with a specific trait. Transcriptome analysis assists in identifying differentially expressed genes (DEGs) and provides an insight into altered cellular processes, signaling pathways and networks. Previously, RNA-seq and microarray-based transcriptome analyses have helped to understand the K^+^ deficiency response in plant species, such as *Arabidopsis thaliana* (Armengaud et al., 2004), rice (Shankar et al., 2013; Zhang et al., 2017), wheat (Zhao et al., 2020), tomato (Zhao et al., 2018), cotton (Yang et al., 2021), pear (Shen et al., 2017; Yang et al., 2020), foxtail millet (*Setaria italica* L.) (Cao et al., 2019) and sweet potato (*Ipomoea batatas*) (Wang et al., 2022). However, no transcriptome base study has been undertaken so far to understand the molecular mechanism of K^+^ deficiency tolerance in chickpea. Therefore, to investigate the K^+^ deficiency responsive transcriptome in PUSA362 and PUSA372 cultivars, RNA-seq analysis was performed under K^+^ sufficient and deficient conditions. About 303 and 307 million raw reads were generated from six samples (three K^+^ sufficient, three K^+^ deficient) of PUSA362 and PUSA372, respectively. Out of these, 299 and 303 million clean reads were obtained in PUSA362 and PUSA372. The Q30 values of ~ 94% for both cultivars indicated that the quality of sequencing data was good and significant. The average alignment rate for PUSA362 and PUSA372 were 91.14% and 87.56% respectively (**Table 1**). The analysis and identification of DEGs was performed using DESeq2 v1.30.1 package. To account for the handling errors and variations among the samples, DEGs with Log_2_ fold change ±1 and a p-value of < 0.05 were considered significant. We found a total of 820 significantly expressed DEGs in PUSA362, out of which 311 genes were up-regulated and 509 were down-regulated (**Figure 3** and **Table S3**). In PUSA372, out of a total of 682 DEGs, 207 genes were up-regulated and 475 genes were down-regulated. Most of the DEGs from both chickpea cultivars could be assigned an NCBI locus ID and annotated with a known function. For some of the DEGs, NCBI locus ID and/or known functional annotations were not available thus, they were classified as DEGs with unknown function. These DEGs are denoted by gene name starting with “MSTRG” (**Table S3**). A global view of all DEG’s in both varieties is presented through a volcano plot (**Figure 4A**). Notably, a significant number of DEGs was found to have overlapping, opposite and unique expression in both the chickpea cultivars. 258 and 159 genes were exclusively up-regulated, respectively in PUSA362 and PUSA372. While 386 and 347 unique genes were exclusively down-regulated in PUSA362 and PUSA372, respectively (**Figure 4B**). Interestingly, 14 genes showed contrasting expression pattern, as they were up-regulated in PUSA362 and down-regulated in PUSA372. These DEGs include somatic embryogenesis receptor-like kinase 1 (LOC113785888), thaumatin-like protein (LOC101511048), ferric reduction oxidase 2 (LOC101499003) and transcription factor bHLH93 (LOC101489960). On the other hand, 9 genes which showed down-regulation in PUSA362 were up-regulated in PUSA372, and these include glutathione S-transferase zeta class-like (LOC113784455), putative calcium-binding protein CML19 (LOC101492172), high affinity nitrate transporter 2.4 (LOC101503875) and ethylene-responsive transcription factor ERF017-like protein (LOC101496587). OA, OB, OC and OD identifiers were used for putative novel genes present in both varieties (**Table S4**). Thus, it is evident from transcriptome analysis that K^+^ deficiency triggered the transcriptional reprogramming in different chickpea cultivars (PUSA362 and PUSA372). Interestingly, more numbers of DEGs were down-regulated than up-regulated in both the chickpea cultivars. This indicates that during short-term K^+^ deficiency chickpea plants might need repression of different cellular processes, like those involved in energy expenditure and metabolism, to support the plant growth. Thus, DEGs involved in key signaling networks in each chickpea cultivars could be responsible for their contrasting morphophysiological response to K^+^ deficiency.

**Table 1:** Summary of sequence assembly data analysis after RNA sequencing.

Sample name	Raw reads	Clean reads	Clean bases	Average coverage (%)	Q20 (%)	Q30 (%)	Alignment rate
PUSA362, KS1	43.791554 M	43.370770 M	6.407773 G	44.3633	97.958291	93.736127	89.34%
PUSA362, KS2	46.498322 M	46.013488 M	6.790540 G	41.4191	98.04188	93.952486	85.64%
PUSA362, KS3	65.731198 M	64.992878 M	9.630451 G	56.935	97.932701	93.660516	89.73%
PUSA362, KD1	57.020970 M	56.359848 M	8.335876 G	57.3107	98.070383	94.103813	93.71%
PUSA362, KD2	44.067446 M	43.570722 M	6.455914 G	46.3386	97.908821	93.617395	93.92%
PUSA362, KD3	45.901854 M	45.364060 M	6.697309 G	46.3855	98.184631	94.367596	94.50%
PUSA372, KS1	46.969232 M	46.360326 M	6.789864 G	40.7649	98.08786	94.228344	82.12%
PUSA372, KS2	54.918072 M	54.367048 M	8.025501 G	55.1703	97.843193	93.46048	93.13%
PUSA372, KS3	46.477222 M	45.713680 M	6.682883 G	39.0247	97.777873	93.608092	90.77%
PUSA372, KD1	48.760200 M	48.237750 M	7.112087 G	46.453	98.081043	94.010408	89.37%
PUSA372, KD2	47.119356 M	46.651980 M	6.920498 G	46.8844	97.921428	93.656282	89.38%
PUSA372, KD3	63.383762 M	62.484308 M	9.173471 G	53.1041	97.942865	93.778872	80.57%

KS – K^+^ sufficient, KD – K^+^ deficient.

### 3.3 Validation of RNA-seq expression data by RT-qPCR

**Figure 5:**
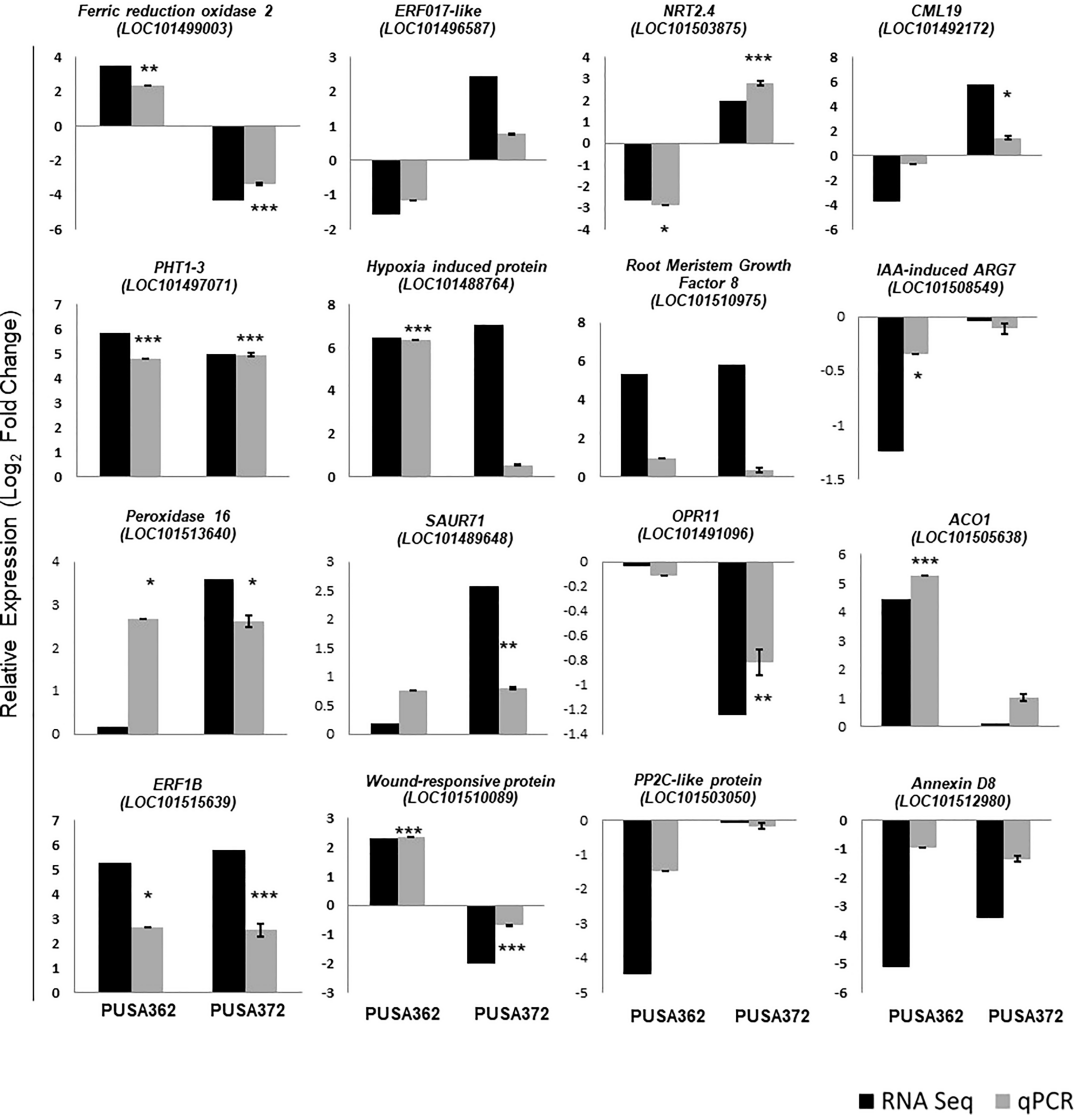
Validation of RNA-seq expression data using RT-qPCR analysis. 16 DEGs were randomly selected and their RNA-seq expression pattern was validated by RT-qPCR both in PUSA362 and PUSA372 cultivars. Each bar represents average of expression data from three biological replicates. X-axis shows the name of chickpea cultivar and Y-axis represents the relative expression in terms of log_2_ fold change. Name of the genes with locus IDs are given on top of the graphs. Standard error was calculated from standard deviation among three biological replicates and is shown on each bar. Asterisks indicates the statistical significance among replicate samples with p-value as *p < 0.05, **p < 0.01 and ***p < 0.005.

To ensure the accuracy of RNA-seq data, the expression pattern of 16 randomly selected genes was verified by RT-qPCR. Out of 16 selected DEGs, 13 showed similar expression pattern by both RNA-seq and RT-qPCR methods in PUSA362 variety (**Figure 5** and **Table S5**). These included genes like ferric reduction oxidase 2 (LOC101499003), high affinity nitrate transporter 2.4 (LOC101503875), probable inorganic phosphate transporter 1-3 (LOC101497071), putative hypoxia induced protein (LOC101488764), 1-aminocyclopropane-1-carboxylate oxidase 1 (LOC101505638) and wound-responsive family protein (LOC101510089). Similarly, in PUSA 372, 13 DEG’s showed similar expression pattern by both, RNA-seq and RT-qPCR analysis. These genes included ferric reduction oxidase 2 (LOC101499003), high affinity nitrate transporter 2.4 (LOC101503875), probable inorganic phosphate transporter 1-3 (LOC101497071), peroxidase 16 (LOC101513640) and putative 12-oxophytodienoate reductase 11 (LOC101491096). In PUSA372 genes like indole-3-acetic acid-induced protein ARG7 (LOC101508549) and phosphatase 2C-like protein 44 isoform X1 (LOC101503050) showed no significant expression by RNA-seq whereas, they were found to be down-regulated by -0.1 and -0.17 Log_2_fold when their expression was analyzed using RT-qPCR. Similar expression pattern of significant proportion of tested genes by both the techniques in both chickpea cultivars make this RNA-seq expression data reliable and significant, and it confirms the differential regulation of chickpea genes under K^+^ deficiency conditions. Thus, RT-qPCR expression analysis largely verified the expression pattern obtained from RNA-seq analysis in both the chickpea cultivars.

### 3.4 Gene ontology-based classification of DEGs

**Figure 6:**
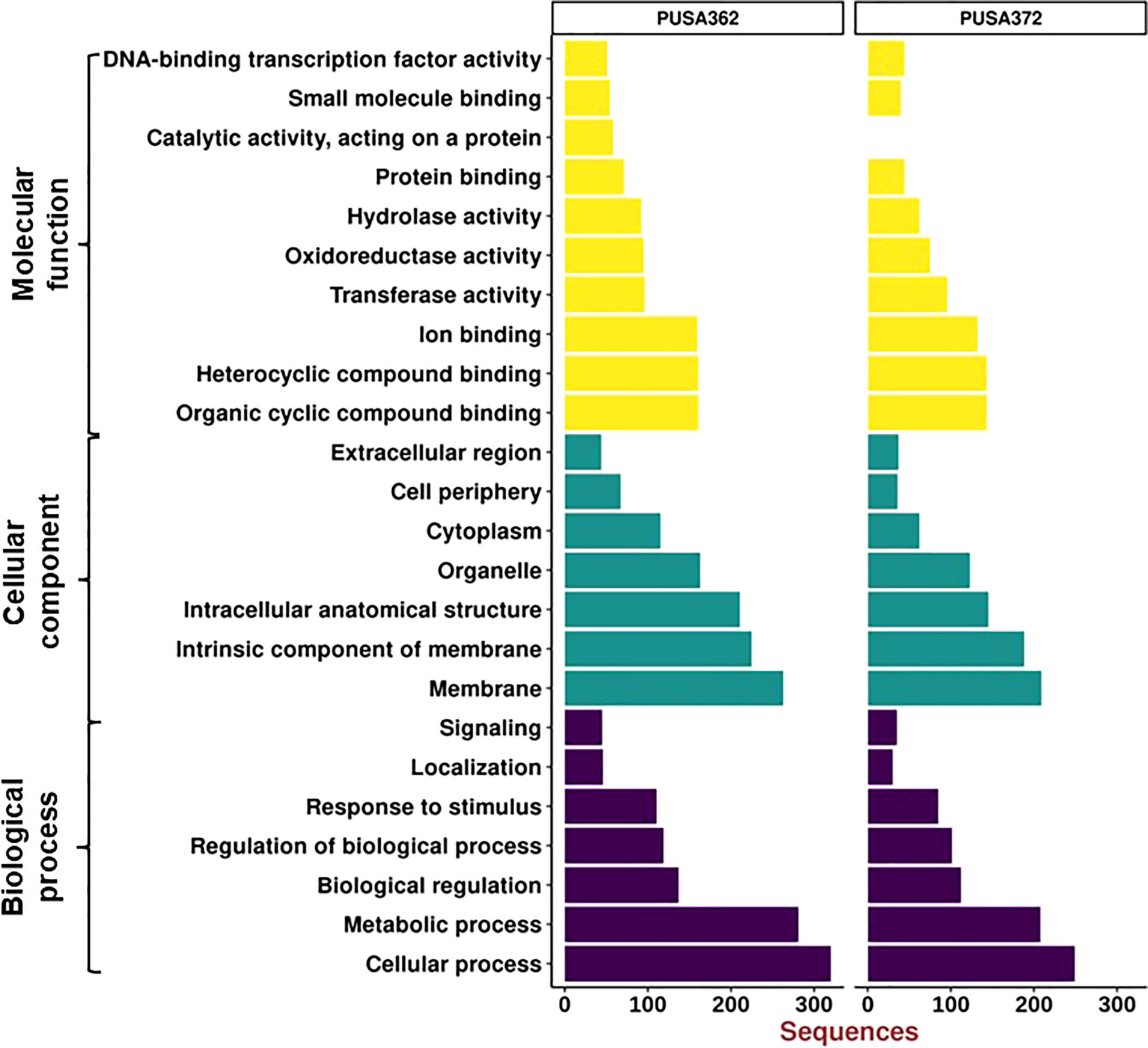
Gene Ontology enrichment analysis of DEGs of PUSA362 and PUSA372 chickpea cultivars. Each DEG’s assigned with at least one GO term and categorized into three main categories and 24 total sub-categories. Three main categories include biological process, cellular component and molecular function. Y-axis indicates GO category name and X-axis shows the number of DEG sequences.

Gene Ontology (GO) enrichment analysis was performed to understand the functional relevance of K^+^ deficiency responsive DEGs in chickpea. GO analysis differentiated DEGs based on their molecular functions, cellular component and biological process. Within these three main categories, a total of 24 sub-categories were identified in PUSA362, and 23 sub-categories in PUSA372 (**Figure 6**). DEGs in molecular functions category could be classified into 10 sub-categories in PUSA362 and into 9 sub-categories in PUSA372. Surprisingly, DEGs with molecular function of catalytic activity or acting on a protein were absent in PUSA372. Other major categories in molecular functions included, organic and heterocyclic compound binding, ion binding, transferase activity, oxidoreductase activity, hydrolase activity and transcription factor (TF) activity. Highest number of DEGs belong to heterocyclic compound binding, organic compound binding and ion binding in both the chickpea cultivars (**Table S6**). A significant number of DEGs (283 in PUSA362 and 233 in PUSA372) were associated with important enzymatic activity, including transferase, oxidoreductase and hydrolase. It has been well known that K^+^ is required for the activation of more than 60 different types of enzymes which are involved in important cellular processes, such as protein synthesis, starch synthesis, osmoregulation and photosynthesis (Hafsi et al., 2014). Association of several DEGs with important enzymatic functions indicates that K^+^ deficiency may have altered the functional behavior and activities of different enzymes that could lead to modulation of important cellular processes in chickpea. In cellular component, DEGs were associated with seven different sub-categories, such as extracellular region, cell periphery, cytoplasm, organelle and cell membrane components. Highest number of DEGs was found to be associated with membrane in both the chickpea cultivars. In PUSA362, a total of 263 membrane associated DEGs were present while, 209 membrane associated DEGs were found in PUSA372. In addition, 225 and 188 DEGs were identified as intrinsic components of membrane in PUSA362 and PUSA372, respectively. This suggests that K^+^ deficiency regulates several membrane associated proteins. These proteins may be involved in important cell membrane related functions, such as stress sensing/perception, membrane structure integrity, membrane lipid remodeling, ion and nutrient transport across membrane etc., in chickpea. In addition, some of the DEGs might be involved in regulating K^+^ concentration around the membranes to maintain membrane potential, as K^+^ is well known for its role in maintenance of membrane potential and electrical neutralization (Wang and Wu, 2013). In biological processes also, DEGs belong to seven sub-categories in both cultivars and these include, cellular processes, metabolic processes, biological pathways, response to stimulus and signaling. DEGs associated with cellular processes were highest both in PUSA362 (320 DEGs) and PUSA372(249 DEGs), followed by metabolic processes, to which 281 and 208 DEGs were associated in PUSA362 and PUSA 372, respectively (**Table S6**). This is again suggestive of the fact that K^+^ deficiency results in modulation of numerous cellular processes and signaling networks in chickpea. Some of these important cellular processes and biological functions are discussed in upcoming sections.

### 3.5 DEGs are associated with different biological pathways

**Figure 7:**
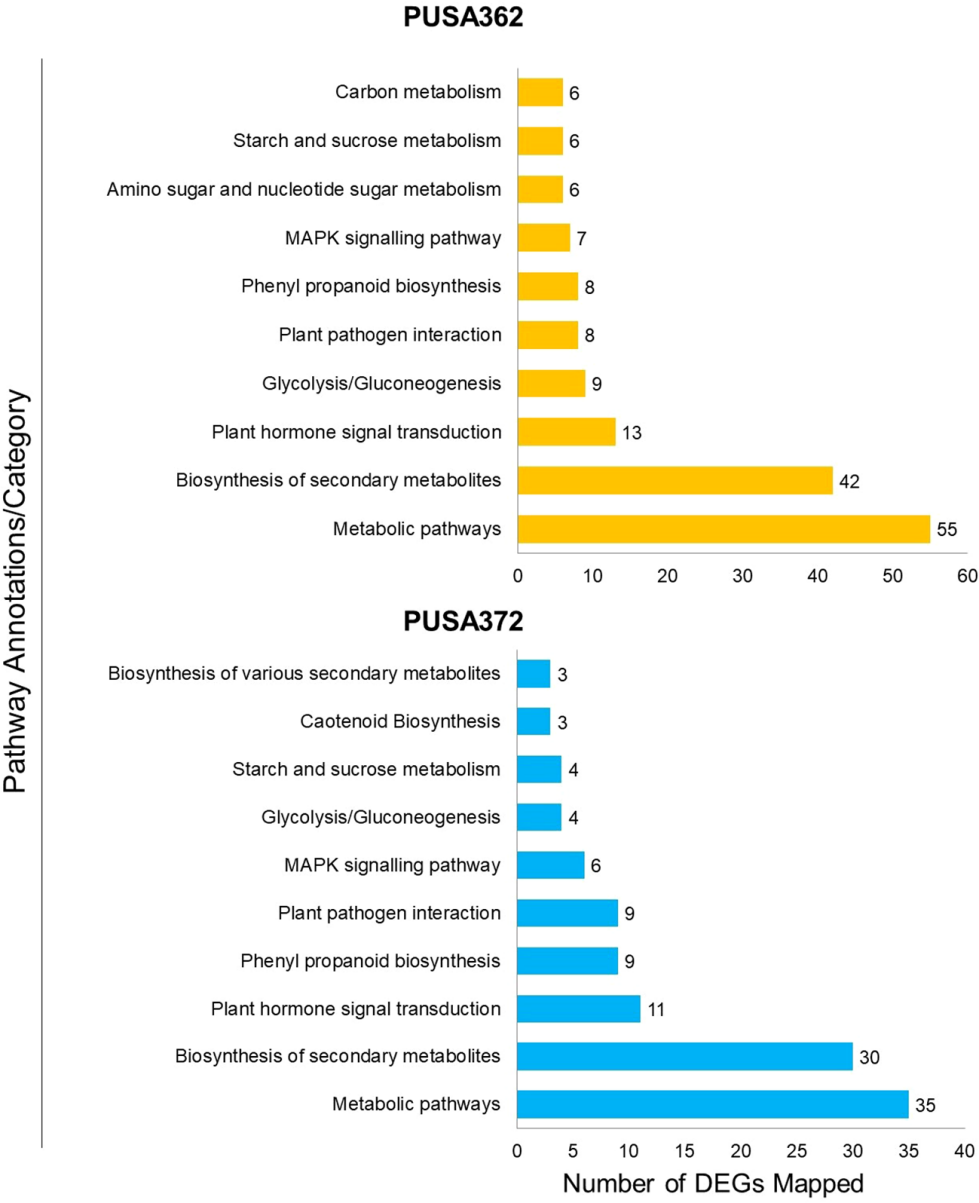
Kyoto Encyclopedia of Genes and Genomes (KEGG) pathway analysis of DEGs of both PUSA362 and PUSA372 chickpea cultivars under K^+^ deficiency. The Y-axis indicates the pathway annotations/category whereas, X-axis shows the total number of DEGs mapped to corresponding pathways in each variety. A number shown against each bar indicate the total number of DEGs belonging to a particular pathway annotation/category.

To understand that which biological pathways are affected in chickpea plant cell by K^+^ deficiency, KEGG (http://www.genome.jp/kegg/) pathway enrichment analysis was performed. KEGG helps to understand the biological function of DEGs by mapping them to the whole genome pathway database. In this analysis, 820 DEGs of PUSA362 were mapped to 65 pathways and 682 DEGs of PUSA372 were mapped to 49 pathways. Highest DEGs were mapped to “metabolic pathways” in both PUSA362 (55 DEGs) and PUSA372 (35 DEGs) (**Figure 7** and **Table S7**). 29 and 13 genes that belonged to “metabolic pathways” were up-regulated while, 27 and 24 genes were down-regulated in PUSA362 and PUSA372, respectively. The pathway where second highest DEGs were mapped was “biosynthesis of secondary metabolites”. In this category, 23 and 11 genes were up-regulated whereas, 21 and 22 genes were down-regulated in PUSA362 and PUSA372, respectively. A number of DEGs was mapped to various other important pathways, such as hormone signal transduction pathway, plant pathogen interaction pathway, mitogen activated protein kinases (MAPK) signaling pathway in both the chickpea cultivars. Interestingly, few genes were found to be mapped to pathways which were cultivar specific, for example, amino sugar and nucleotide sugar metabolism and carbon metabolism in PUSA362 whereas, carotenoid biosynthesis in PUSA372. Overall, GO and KEGG pathway analysis of DEGs indicate that chickpea plants respond to K^+^ deficiency through alteration in metabolism, gene expression, phytohormone signaling, ion transport activity, signal transduction, RSA and root growth.

#### 3.5.1 K+ deficiency responsive metabolism related genes

Analysis revealed that about 74 DEGs in PUSA362 and 54 DEGs in PUSA372 were associated with plant metabolism. These DEGs belonged to various components of plant metabolism including metabolic pathways, secondary metabolites biosynthesis, pyruvate metabolism, glycolysis/gluconeogenesis, carbon metabolism, amino sugar and nucleotide sugar metabolism, starch and sucrose metabolism. These key genes are known to be involved in energy production, plant growth and development. K^+^ serves as a co-factor for numerous important metabolic enzymes and involved in metabolite transport as a counter ion. Thus, K^+^-deficiency could result in serious metabolic disorders in plants (Armengaud et al., 2009). Deficiency of K^+^ has been found to alter important metabolic processes and pathways in Arabidopsis and rice, including carbohydrate metabolism, the TCA cycle, organic acid metabolism and amino acid metabolism (Armengaud et al., 2004; Shankar et al., 2013). Consistently, genes such as fructose-1,6-bisphosphatase (LOC101500563), UTP-glucose-1-phosphate uridylyltransferase (LOC101490076) and sucrose synthase (LOC101514095) which are involved in carbohydrate metabolism, glycolysis/gluconeogenesis and sucrose metabolism (Tamoi et al., 2006; Stein and Granot, 2019) were significantly down-regulated in K^+^ sensitive PUSA362 chickpea. Suppression of such important genes could severely hamper the photosynthesis rate, sucrose production, and it supply to the chickpea roots. Limited availability of important carbon source sucrose may have led to energy starvation condition, and consequently retarded root and plant growth in PUSA362. Similarly, genes like phosphoserine phosphatase (LOC113786736) and cyanogenic beta-glucosidase (LOC101506343) which have crucial role in metabolism and defense against plant herbivores (Lai et al., 2015; Samuilov et al., 2018) were down regulated by -8 and -5 Log_2_ fold, respectively in PUSA362. Uridine diphosphate (UDP)-glycosyltransferase (UGT) members (LOC101514581, LOC101514447) were specifically up-regulated by 3 Log_2_ fold change in PUSA372 but they were not significantly expressed in PUSA362. Similarly, a UGT encoding gene was also found to be down-regulated in K^+^ sensitive rice variety IR64 (Shankar et al., 2013). UGTs are known to function in metabolic pathways, secondary metabolite biosynthesis and have role in homeostasis and detoxification (Huang et al., 2021; Kulasekaran et al., 2021; Mateo-Bonmatí et al., 2021). This indicate that in addition to primary metabolism, K^+^ deficiency may have negatively affected the secondary metabolite synthesis and homeostasis in PUSA362 but not in PUSA372. Interestingly, a UGT gene was also expressed differentially under iron toxicity in rice (Quinet et al., 2012), suggesting that UGT are involved in metabolic processes which are commonly affected due to variable availability of different nutrients. Other important genes which are involved in symbiotic nodule formation e.g., early nodulin-like protein (LOC101510639) and those involved in plant energy production process *via* electron transport chain e.g., NADH dehydrogenase (ubiquinone) (LOC113786610) showed more than 4 Log_2_ fold up-regulation in PUSA362 (**Table 2**). Probable xyloglucan endotransglucosylase (LOC101490426), polygalacturonase (LOC101508055) and glucan endo-1,3-beta-glucosidase (LOC101509535) which are important components of xyloglucan metabolism and involved in cell wall assembly and growth (Babu and Bayer, 2014; Hara et al., 2014) were induced significantly in PUSA362. The cell wall is crucial for maintenance of cell structure and integrity and important barrier in plant defense against biotic factors, such as pathogens (Amtmann et al., 2008). As mentioned earlier, K^+^ deficiency can weaken the plant defense and make plants prone to pathogens. Thus, in order to strengthen the defense, K^+^ sensitive PUSA362 chickpea plant may want to maintain its cell wall *via* induction of cell wall associated genes. Possibly due to same reason, cell wall associated genes have been found to be induced in response to Low-K^+^ stress in other plants, such as Arabidopsis (Armengaud et al., 2004), rice (Shankar et al., 2013) and sweet potato (Wang et al., 2022).Overall, alteration in expression many crucial metabolism related genes may have affected the primary metabolism particularly, carbohydrate metabolism, secondary metabolism, defence related metabolism and plant respiration, variably in PUSA362 and PUSA372.That might have resulted in diverged energy production, plant growth and development under K^+^ deficiency in K^+^ sensitive and tolerant chickpea cultivars.

**Table 2:** Details of some important K^+^ deficiency responsive DEGs in PUSA362 and PUSA372 chickpea cultivars.

Gene LocusID	Function Description	Log2 fold change	Expression regulation
Metabolism related DEGs			
PUSA362			
LOC113786610	NADH dehydrogenase [ubiquinone] iron-sulfur protein 7,	4.4	Up
LOC101506343	cyanogenic beta-glucosidase	-5.6	Down
LOC101500563	fructose-1,6-bisphosphatase, cytosolic	-1.3	Down
LOC101514095	Sucrose synthase	-1.2	Down
PUSA372			
LOC101508799	putative glucose-6-phosphate 1-epimerase	2.7	Up
LOC101514581	UDP-glycosyltransferase 1	1.9	Up
LOC101489836	Alcohol dehydrogenase-like 1	-1.1	Down
LOC101506967	alpha-amylase	-1.4	Down
LOC113784595	patatin-like phospholipase	-5.4	Down
LOC101496167	seed linoleate 9S-lipoxygenase	-1.5	Down
Transcription factors			
PUSA362			
LOC101515639	Ethylene-responsive transcription factor 1B	5.2	Up
LOC101512924	Ethylene-responsive transcription factor ERF098	-2.5	Down
LOC101513362	Ethylene-responsive transcription factor RAP2-3	3.3	Up
LOC101491698	transcription factor MYB14-like	-5.6	Down
LOC101507291	transcription factor bHLH92	-5.1	Down
LOC101491267	MADS-box transcription factor 23	-4.6	Down
LOC101492395	homeobox-leucine zipper protein ATHB-40	-2.8	Down
LOC101508034	LOB domain-containing protein 41	2.2	Up
PUSA372			
LOC101499805	ethylene-responsive transcription factor ERF109	5.9	Up
LOC101515639	ethylene-responsive transcription factor 1B	5.7	Up
LOC101496258	ethylene-responsive transcription factor erf 017-like	3.7	Up
LOC101496910	ethylene-responsive transcription factor erf017-like	3.4	Up
LOC101496811	AP2-like ethylene-responsive transcription factor	-3.7	Down
LOC105851404	transcription factor MYB46	-5.0	Down
LOC101503635	transcription factor MYB98	-5.4	Down
LOC101501101	transcription factor bHLH146	-1.6	Down
LOC101492395	homeobox-leucine zipper protein ATHB-40	-1.3	Down
LOC101504817	LOB domain-containing protein 12	2.3	Up
LOC101490093	WUSCHEL-related homeobox 9	5.0	Up
Signal transduction components			
PUSA362			
LOC101507945	CBL-interacting protein kinase 2	1.2	Up
LOC113785888	somatic embryogenesis receptor-like kinase 1	5.1	Up
LOC101510368	putative receptor-like protein kinase At4g00960	4.9	Up
LOC101504201	probable serine/threonine-protein kinase fhkB	2.7	Up
LOC101508412	probable protein phosphatase 2C 25	1.2	Up
LOC101493828	probable protein phosphatase 2C 15	-5.8	Down
LOC101503050	phosphatase 2C-like protein 44 isoform X1	-4.5	Down
LOC101500357	type I inositol polyphosphate 5-phosphatase 5	-3.8	Down
LOC101492388	calmodulin-binding receptor-like cytoplasmic kinase 2	-1.1	Down
LOC101512980	annexin D8	-5.1	Down
LOC101492172	putative calcium-binding protein CML19	-3.7	Down
LOC101507335	probable calcium-binding protein CML41	1.6	Up
LOC101501436	calcium-binding protein PBP1	1.3	Up
LOC101496110	cationic peroxidase 1	3.1	Up
LOC113784455	glutathione S-transferase zeta class-like	-5.3	Down
PUSA372			
LOC101493042	receptor-like cytosolic serine/threonine-protein kinase RBK1	1.1	Up
LOC101502405	inorganic pyrophosphatase 2-like	1.4	Up
LOC101513274	probable protein phosphatase 2C 72	1.4	Up
LOC101515110	acid phosphatase 1	1.4	Up
LOC113784622	type I inositol polyphosphate 5-phosphatase 12, X2	-5.3	Down
LOC113785888	somatic embryogenesis receptor-like kinase 1	-5.2	Down
LOC101499928	CBL-interacting serine/threonine-protein kinase 1, X1	-1.1	Down
LOC101512278	1-aminocyclopropane-1-carboxylate oxidase homolog 1-like	-1.5	Down
LOC101495667	cysteine-rich receptor-like protein kinase 10 isoform X2	-1.1	Down
LOC101492172	putative calcium-binding protein CML19	5.7	Up
LOC101515597	calcium-binding protein PBP1-like	2.6	Up
LOC101512980	annexin D8	-3.4	Down
LOC101513640	Peroxidase 16	3.6	Up
LOC101491218	peroxidase P7-like	2.1	Up
LOC101489588	probable glutathione S-transferase	-1.2	Down
Ion transport associated DEGs			
PUSA362			
LOC101497071	probable inorganic phosphate transporter 1-3	5.8	Up
LOC105851115	protein NRT1/PTR FAMILY 3.1-like	1.6	Up
LOC101512428	vacuolar iron transporter homolog 4-like	-4.4	Down
LOC101503875	high affinity nitrate transporter 2.4	-2.6	Down
LOC101506906	high affinity nitrate transporter 2.5	-1.2	Down
LOC101497328	protein NRT1/PTR FAMILY 5.6-like	-1.2	Down
LOC101497004	protein NRT1/PTR FAMILY 2.6	-1.1	Down
LOC101495372	S-type anion channel SLAH1	-1.5	Down
PUSA372			
LOC101503875	high affinity nitrate transporter 2.4	1.9	Up
LOC101497071	probable inorganic phosphate transporter 1-3	4.9	Up
LOC101495372	S-type anion channel SLAH1	-1.9	Down
LOC101497328	protein NRT1/PTR FAMILY 5.6-like	-1.4	Down
LOC101504169	bidirectional sugar transporter SWEET3	1.5	Up
Phytohormones associated DEGs			
PUSA362			
LOC101505638	1-aminocyclopropane-1-carboxylate oxidase 1	4.4	Up
LOC101489126	auxin-responsive protein SAUR72-like	2.8	Up
LOC101513626	gibberellin 2-beta-dioxygenase	1.1	Up
LOC101509106	cytochrome P450 94C1	2.2	Up
LOC101504297	cytochrome P450 71D10-like	-1.4	Down
LOC113787771	3-epi-6-deoxocathasterone 23-monooxygenase	-1.5	Down
PUSA372			
LOC101489648	Auxin-responsive protein SAUR71	2.6	Up
LOC101489126	auxin-responsive protein SAUR72-like	2.4	Up
LOC101500571	gibberellin 20 oxidase 2-like	-2.7	Down
LOC101512278	1-aminocyclopropane-1-carboxylate oxidase homolog 1-like	-1.1	Down
LOC101510386	auxin-responsive protein SAUR50-like	-1.3	Down
LOC101501719	auxin-responsive protein SAUR68-like	-1.3	Down
DEGs associated with root growth and development			
PUSA362			
LOC101505027	expansin-A11	1.2	Up
LOC101513147	patatin-like phospholipase	5.7	Up
LOC113786110	protein MAIN-LIKE 1-like	4.6	Up
PUSA372			
LOC101500741	putative expansin-B2	7.2	Up
LOC101504687	putative expansin-A30	1.2	Up
LOC113784595	patatin-like phospholipase	-5.4	Down
LOC113786110	protein MAIN-LIKE 1-like	-5.0	Down

#### 3.5.2 K^+^ deficiency related transcription factors in chickpea cultivars

**Figure 8:**
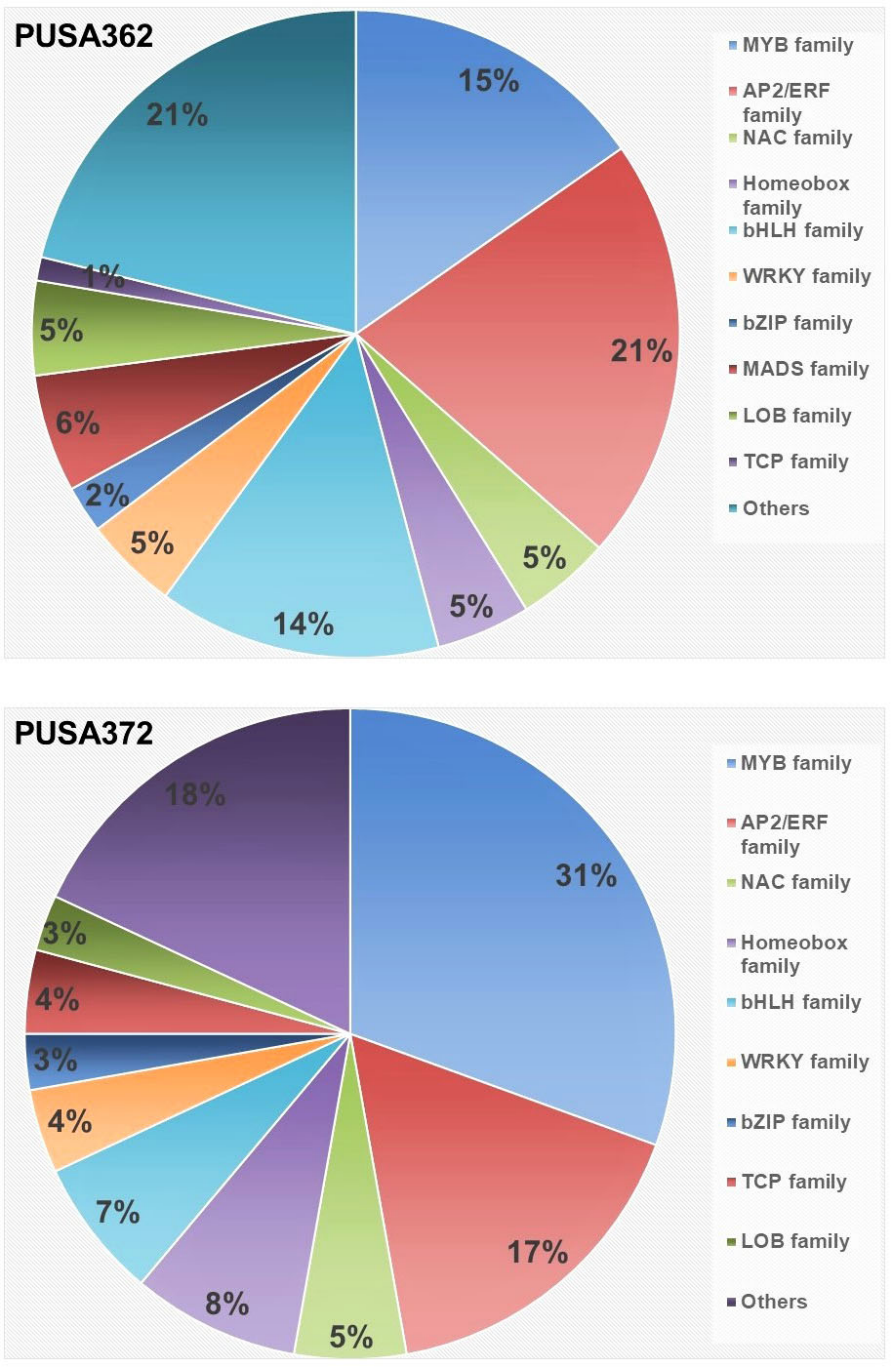
Distribution of K^+^ deficiency responsive, differentially expressed transcription factor in PUSA362 and PUSA372 cultivars. Color legends and corresponding names of different transcription factor family are denoted on the right. Numbers in the different color compartments of pi-chart indicate total TF from different family as DEGs.

Transcription factors (TFs) play important regulatory roles in several plant processes, including response to stresses, such as K^+^ deficiency. In depth analysis of our transcriptome data revealed that several DEGs belong to TFs in both chickpea cultivars. In PUSA362, a total of 86 DEGs were found to encode TFs and “regulation of transcription” related proteins. Out of 86 DEGs, 28 were up-regulated and 58 were down-regulated. Similarly, 72 TF encoding DEGs were found in PUSA372, out of which 17 were up-regulated and 55 were down-regulated (**Table S3**). In PUSA362, most differentially expressed TF were from APETALA2 (AP2)/Ethylene Responsive Factor (ERF) (21%) and MYB (15%) family. Similarly, in PUSA372, most differentially expressed TFs belong to MYB (31%) and AP2/ERF (17%) TF family. TFs of basic helix–loop–helix (bHLH) family in PUSA372 were half (7%) compared with PUSA362 (14%). Other important differentially expressed TFs includes NAC, WRKY, bZIP and Homeobox family proteins in both chickpea cultivars (**Figure 8** and **Table S8**). TFs belonging to AP2, MYB, ARF, bHLH and WRKY family have been found to be differentially expressed in response to K^+^ deficiency in Arabidopsis (Armengaud et al., 2004) and crop plants such as rice (Shankar et al., 2013), wheat (Zhao et al., 2020), foxtail millet (Cao et al., 2019) and sweet potato (Wang et al., 2022). This indicates that these TFs have a conserved role in transcriptional reprogramming of different genes under K^+^ deficiency in chickpea and other plants. TFs of AP2/ERF family are known to play an important role in regulating K^+^ deficiency response in plants. Arabidopsis RAP2.11 (AP2/ERF) binds to the GCC box in the promoter of *High Affinity K^+^ Transporter 5* (*HAK5*) and regulates its expression under K^+^ deficient conditions through reactive oxygen species (ROS) dependent pathway (Kim et al., 2012; Schachtman, 2015). TFs of auxin responsive factor (ARF) family have also been implicated in K^+^ deficiency response. AtARF2 represses the *AtHAK5* expression under K^+^ sufficient (normal) conditions while, under K^+^ deficient conditions AtARF2 is phosphorylated, which relieves the repression from AtHAK5, which in turn, mediates enhanced K^+^ uptake (Zhao et al., 2016). NITRATE TRANSPORTER 1/PEPTIDE TRANSPORTER FAMILY 7.3 (NPF7.3) is a proton coupled H^+^/K^+^ antiporter which mediates root-to-shoot K^+^ translocation. AtMYB59 binds to the promoter of NPF7.3 and positively regulates its expression under external K^+^/NO_3_^–^application (Du et al., 2019). AtMYB77 is involved in auxin signal transduction, and its expression is reduced under K^+^ deficiency to regulate lateral root development (Shin et al., 2007). Importantly, AtMYB77 binds to AtHAK5 promoter and positively regulates its expression to improve K^+^ uptake by roots (Feng et al., 2021). Similarly, bHLH122 and WRKY33 TFs regulate the expression of an Arabidopsis K^+^ transporter, K^+^ UPTAKE 2(KUP2) (Rajappa et al., 2020). Like Arabidopsis, these important TFs could be involved in regulating expression of K^+^ transporters, such as HAKs, KUP and NPF7.3 in chickpea, and may contribute to enhanced K^+^ uptake, root-shoot translocation and redistribution under K^+^ deficient conditions. In addition, TFs could also regulate the expression of development related genes, such as those involved in root growth.

#### 3.5.3 K^+^ deficiency related signal transduction components

High amount of ROS is known to be accumulated under K^+^ deficiency, and it interferes in the normal function of proteins and lipids which leads to cell damage (Mittler, 2017). Although, accumulation of ROS may lead to oxidative stress that can be damaging to cells, ROS is known as an important signaling molecules. Therefore, in plant cell several proteins function to strike a balance between ROS production and detoxification (Sagi and Fluhr, 2006; Vellosillo et al., 2010; Sharma et al., 2012). Peroxidases and glutathione S-transferase (GST) are involved in this process and both act as ROS scavengers. In Arabidopsis roots, the expression of a peroxidase encoding gene *ATP19a* was significantly reduced under K^+^ deficiency (Armengaud et al., 2004). In rice, the expression of GST encoding genes was found to be down-regulated under K^+^ deficiency (Shankar et al., 2013). Similarly, several peroxidase and GST genes were differentially expressed in wheat under K^+^ deficiency conditions. In this study, several members of peroxidase family and GSTs were found to be differentially expressed in both chickpea cultivars. For example, GST encoding gene LOC113784455 showed -5.29 Log_2_ fold down-regulation in PUSA362 and 3.08 Log_2_ fold up-regulation in PUSA372. This infers that like in other plants, peroxidase and GST *via* ROS scavenging regulate the ROS accumulation and homeostasis to prevent the chickpea plants from oxidative damage due to low-K^+^ stress.

K^+^ deficiency is known to trigger Ca^2+^ signaling in plants. Several Ca^2+^ signaling components, such as calmodulin (CaM), calcineurin B-like (CBL), CBL-interacting protein kinases (CIPKs) have been found to be induced under K^+^ deficiency and regulating K^+^ deficiency response in plants (Armengaud et al., 2004; Lee et al., 2007; Pandey et al., 2007; Singh et al., 2018). In this study, a CIPK gene (LOC101507945) was up-regulated in PUSA362 whereas, another CIPK member (LOC101499928) was significantly down-regulated in PUSA372. In depth analysis revealed that more phosphorylation related genes are up-regulated in PUSA362 than in PUSA372. Genes encoding protein phosphatase 2C (PP2C) (LOC101493828, LOC101503050) were down-regulated by -5.8 and -4.4 Log_2_ fold in PUSA362. In PUSA372, type I inositol polyphosphate 5-phosphatase was found to be down-regulated by -5.8 Log_2_ fold (**Table 2**). This indicates that besides transcriptional regulation, post-translational regulation of genes is critical of K^+^ deficiency tolerance in chickpea. It is well known that signaling components, such as kinases and phosphatases play an important role in regulating K^+^ uptake and transport in plants. Primarily, kinases such as CIPKs and phosphatases of PP2C family post-translationally regulate K^+^ transporters and channels like HAK5 and Arabidopsis K^+^ transporter 1 (AKT1) in response to K^+^ deficiency (Singh et al., 2016; Singh et al., 2018; Ankit and Singh, 2022). CIPK23 in complex with CBL1 and CBL9 phosphorylates and activates AKT1, to enhance root K^+^ uptake under K^+^ deficient conditions (Lee et al., 2007). Also, CIPK6 interacts with CBL4 and regulate the translocation of K^+^ channel AKT2 to plasma membrane, that ultimately enhance AKT2 activity (Held et al., 2011; Singh et al., 2016). In addition, Ca^2+^ sensor proteins such as CBLs have been implicated in K^+^ deficiency response. For example, CBL2 and CBL3 which interact with four CIPKs (CIPK3, 9, 23 and 26) in Arabidopsis, regulate K^+^ homeostasis through activating TPK (two-pore K) channel mediated vacuolar K^+^ efflux to the cytoplasm (Tang et al., 2020). PP2C-type phosphatase like AKT1 INTERACTING PROTEIN PHOSPHATASE 1 (AIP1) dephosphorylate AKT1 and functions as a negative regulator of AKT1 channel activity under K^+^ deficiency (Lee et al., 2007; Singh et al., 2015). Similarly, AP2C1 (PP2C), interacts with and dephosphorylates an autophosphorylated CIPK9 (a positive regulator of K^+^ deficiency tolerance), thus, its function as a negative regulator of K^+^ deficiency tolerance in Arabidopsis (Singh et al., 2018). AtPP2CA which is involved in ABA signaling, inhibits gated outwardly-rectifying K^+^ (GORK) channels K^+^ efflux activity independent of its phosphorylation status (Lefoulon et al., 2016). Here, we found that PP2Cs; LOC101493828, LOC101503050 were strongly down-regulated in PUSA362 whereas, five other phosphatases (LOC101502405, LOC101515110, LOC101513274, LOC101504848 and LOC113785826) were significantly up-regulated in PUSA372. We also found genes encoding calcium uniporter, Annexin D, Ca^2+^ binding proteins, like PINOID-BINDING PROTEIN (PBP), CaM-like (CML), CaM binding protein (CBP), etc. as DEGs in both chickpea cultivars. One calcium uniporter (LOC101508599) was found to be up-regulated in PUSA362. While, Annexin D4 and D8 were down-regulated in both, PUSA362 (-5.1 and -1.3 Log_2_fold) and PUSA372 (-3.4 and -1.5 Log_2_fold) (**Table 2**). Thus, signaling components like CBL, CaM, CML, CIPK and PP2C may regulate various K^+^ transporters and channels *via* Ca^2+^ signaling, to improve K^+^ uptake and transport in chickpea, thereby contribute to better KUE. Overall, these findings infer that like in other plants, a conserved CBL-CIPK-PP2C signaling module might be involved in regulating K^+^ uptake and transport in chickpea.

#### 3.5.4 K^+^ deficiency responsive ion/nutrient transport related genes

K^+^ deficiency could have disturbed the homeostasis of different ions and nutrients as several ion/nutrient transporters were identified as DEGs in both chickpea cultivars. These DEGs belonged to ABC transporters, metal transporters, anion channels, transporters for nitrate, phosphate and zinc. An important inorganic phosphate (Pi) transporter (LOC101497071) was up-regulated by 5.8 and 4.97 Log_2_ fold in PUSA362 and PUSA372, respectively (**Table 2**). Members of dual-affinity nitrate transporter/peptide transporter (NRT1/PTR) family were differentially expressed in both the cultivars. High affinity nitrate transporters, LOC101503875 and LOC101506906 were down-regulated in PUSA362 whereas, LOC101503875 was up-regulated significantly in PUSA372. Different members of S-type anion channel (SLAH1) family were down-regulated in both varieties. One of the vacuolar iron transporters homolog 4-like (LOC101512428) was down-regulated specifically in PUSA362 by -4.3 Log_2_ fold. The flux of different ions, metals or solutes is regulated to maintain their homeostasis, osmoticum in the cell and neutralization of the charges across cell membrane. Under K^+^ deficiency, these physiological processes are disturbed and plant cell tends to normalize them *via* different transporters and channels (Walker et al., 1996; Gierth and Mäser, 2007). Thus, differential regulation of several genes encoding ion/metal/solute transporter/channels might be required to maintain the ion homeostasis and membrane potential under K^+^ deficiency in chickpea. Carbohydrate transporter SWEET proteins encoding genes LOC101488880 and LOC101497351 were up-regulated in PUSA362 and PUSA372, respectively. SWEET transporters function as uniporters and mediate sugar diffusion across cell membranes. They mediate sucrose efflux from phloem parenchyma into the phloem apoplasm thus, they are involved in phloem loading (Chen, 2014). As discussed earlier, we found evidence of significant modulation of carbohydrate metabolism under K^+^ deficiency in chickpea. These SWEET transporters could be an important part of the carbohydrate metabolism process. They may help to maintain a sustained supply of sucrose from source to sink for energy production in chickpea under low-K^+^ conditions. In Arabidopsis, most of the K^+^ uptake occur through AKT1, HAK5 and KUP7 transporters (Ankit et al., 2022). The expression level of these transporters in different plants has been found to increase in response to K^+^ deprivation (Ankit and Singh, 2022). Surprisingly, in this study we did not find these transporters genes as DEGs in chickpea. NRT1/PTR members which are found as DEGs here, are known to be involved in nitrate uptake through roots. However, *NRT1/PTR* expression levels is dependent on the external K^+^ level in many plants (Ma et al., 2012; Ruan et al., 2015; He et al., 2020; Yang et al., 2020). K^+^ and NO_3_^-^ have been shown to be coupled during root uptake and transport towards shoots (Ruffel, 2018). NRT1.5/NPF7.3 functions as a proton-coupled H^+^/K^+^ antiporter which can mediates root-to-shoot K^+^ translocation in Arabidopsis (Li et al., 2017). Another Arabidopsis nitrate transporter NRT1.1/NPF6.3 coordinates K^+^ uptake and its translocation from root towards shoots (Bouguyon et al., 2016). This suggest that in chickpea, instead of traditional HAK/AKT/KUP mediated K^+^ transport, a co-transport mechanism of K^+^ and NO_3_^-^ may regulate K^+^ uptake and transport. Importantly, ion transporters may not always be regulated transcriptionally. They might also be regulated post-translationally *via* reversible phosphorylation mechanism by kinases and phosphatases. As discussed earlier, we found several kinases and phosphatases as DEGs which is evidence of significant amount of reversible phosphorylation (post-translational modifications) under K^+^ deficiency in chickpea. However, detail functional investigations are required to establish these assumptions, and to completely understand the K^+^ transport mechanism in chickpea.

#### 3.5.5 K^+^ deficiency responsive phytohormones biosynthesis and signaling genes

Transcriptome data analysis revealed that K^+^ deficiency resulted in modulation of various phytohormones pathways in chickpea. Several genes associated with phytohormone biosynthesis and signaling were found to be differentially expressed in both chickpea cultivars under K^+^ deficiency. In PUSA362, total 44 DEGs were associated with phytohormone, out of which 15 were up-regulated and 29 were down-regulated. In PUSA372, out of total 32 phytohormone related DEGs, only 5 genes were up-regulated and 28 were down-regulated. Ethylene is one of the crucial and early responsive phytohormones to K^+^ deficiency. Ethylene biosynthesis genes are readily induced under K^+^ deficiency and ethylene content is significantly increased as early as 6 hours of K^+^ deficiency in Arabidopsis. Ethylene regulates plants response to K^+^ deficiency, including HAK5 expression, ROS production and primary root growth inhibition (Jung et al., 2009; Schachtman, 2015). We found that a gene encoding for important ethylene biosynthesis enzyme, 1-aminocyclopropane-1-carboxylate oxidase 1 (ACO1) (LOC101505638) was up-regulated by 4.44 Log_2_ fold change in PUSA362 (**Table 2**). Interestingly, an ACO member (LOC101512278) was down-regulated in PUSA372. It is well known that external addition of ethylene or auxin inhibits the primary root growth and root hair elongation, a phenotype similar to one observed in K^+^ deficient plants (Jung et al., 2009; Muday et al., 2012). Importantly, K^+^ deficiency induced inhibition of primary root growth could be reversed in mutants insensitive to auxin (Cao et al., 1993). Interestingly, evidence suggest that ethylene and auxin may function together or cross-talk to regulate root growth development either synergistically or antagonistically (Muday et al., 2012). Also, K^+^ deficiency induced ethylene signal supports auxin biosynthesis and its transport to roots, where auxin accumulates in root elongation zone to inhibit primary root growth and to enhance root hair elongation (Muday et al., 2012; Wang and Wu, 2013). This indicates that ethylene and auxin are crucial in determining the root morphology and RSA under K^+^ deficiency. We also found the evidence of involvement of auxin signaling in K^+^ deficiency response in chickpea. Small auxin up-regulated RNA (SAUR) is one of the largest families of early auxin responsive genes in higher plants (Ren and Gray, 2015). In PUSA362, many SAUR genes (LOC101489126, LOC101509958, LOC101489202, LOC101488509, and LOC101492206) were significantly up-regulated while, some other genes related to auxin response and signaling (LOC101496738, LOC101494568, LOC101508549, LOC101505229, LOC101510386) were significantly down-regulated. Similarly, in PUSA372 auxin responsive SAUR protein encoding genes (LOC101489648, LOC101489126 and LOC101489202) were up-regulated whereas, other auxin signaling and response genes (LOC101508549, LOC101510386, LOC113785858 etc.) were down-regulated. Brassinosteroids (BRs) are also involved in root growth and development in plants (Wei and Li, 2016). Recent transcriptome study showed that various BR related genes were differentially expressed under K^+^ deficient conditions in cotton, and they were implicated in cotton root growth (Yang et al., 2021). In this study, many BR biosynthesis and signaling genes were found to be differentially expressed in both cultivars. BR biosynthesis genes like 3-epi-6-deoxocathasterone23-monooxygenase were down-regulated in both varieties. BR homeostasis related gene cytochrome P450 85A (LOC101510551) was up-regulated, and one beta-amyrin 11-oxidase (LOC101495501) was down-regulated in PUSA362. Like BR, jasmonic acid (JA)has been known to regulate various facets of root growth. It acts as a negative regulator of primary root growth (Chen et al., 2011; Kamali and Singh, 2022), and positive regulator of lateral root (Cai et al., 2014) and root hair growth (Han et al., 2020). JA biosynthesis and signaling genes have been shown to induced in response to K^+^ deficiency in different plants (Armengaud et al., 2004; Li et al., 2017; Deepika and Singh, 2021; Deepika et al., 2022). In this study, JA related gene (LOC101509106) was significantly up-regulated whereas, JA biosynthesis gene (LOC101491096) was down-regulated in PUSA362. Also, various JA catabolism related cytochrome p450 family members were differentially expressed in response to K^+^ deficiency, for example, in PUSA362, LOC101498230, LOC101504297 and in PUSA372, LOC101498230, LOC101504297 were down-regulated. This indicates that K^+^ deficiency stimulated JA accumulation in chickpea is controlled by fine-tuning of JA biosynthesis and catabolism related genes. Variable expression of these genes and accumulation of physiologically significant level of JA may influence the root growth and RSA in chickpea. Besides, gibberellin related gibberellin 2-beta-dioxygenase (LOC101513626) and putrescine related ornithine decarboxylase (LOC101494284) genes were significantly up-regulated in PUSA362. While, genes such as gibberellin 20 oxidase (LOC101492441, LOC101500571, and LOC101491320), genes related to abscisic acid (ABA) (LOC101506315, LOC101505927, LOC101490679) and cytokinin (LOC101495234, LOC101498288) were-down-regulated in PUSA372. Identification of ethylene, auxin, GA, ABA, BR and JA biosynthesis and signaling genes as DEGs under K^+^ deficiency suggests that differential regulation of these genes and reprogramming of these phytohormones pathway could be essential for root growth, RSA changes and overall plant growth in chickpea. Variable expression of these important genes could be one of the reasons of the contrasting growth pattern of two chickpea cultivars under K^+^ deficiency.

#### 3.5.6 K^+^ deficiency responsive genes associated with root growth and development

Our morphological analysis revealed that K^+^ deficiency results in inhibition of primary and lateral root growth in chickpea. It’s expected that the expression of root growth and development related genes might have altered under K^+^ deficiency in chickpea. Consistently, EXPANSIN genes were found to be up-regulated in both chickpea varieties. Expansin family proteins are known to catalyze long-term expansion of cell walls, thus they regulate cell expansion in plants particularly in processes like primary root elongation and root growth (Lee et al., 2003). In PUSA362, EXPANSIN (LOC101505027) was up-regulation by 1.1 Log_2_fold whereas, in PUSA372 EXPANISN (LOC101504687 and LOC101500741) were up-regulated by 1.2 and 7.2 Log_2_fold (**Table 2**). In contrast, LOC101489892 was down-regulated by -1.4 Log_2_fold in PUSA362, whereas no EXPANSIN gene was down-regulated in PUSA372. A leucine-rich repeat (LRR)- extensin (LRX) gene (MSTRG.17226) showed up-regulation by 5.5 Log_2_fold in PUSA362 while, another member of same family (LOC101515776) was down-regulated by -3.4 Log_2_fold in PUSA372.Extensins are the important constituents of the plant cell wall and are involved in cell wall remodeling during cell expansion (Herger et al., 2019). In Arabidopsis, two members of LRX family; *LRX1* and *LRX2* express mainly in root hairs. The *lrx1* mutant was shown to develop root hairs that were deformed and frequently burst. Interestingly, the *lrx2* mutant develops root hairs similar to wild type, however, the *lrx1lrx2* double mutant show more severe phenotype than *lrx1* in terms of hampered root hair development (Baumberger et al., 2001). This indicates synergistic interaction of LRX1 and LRX2 in root hair development. In addition to root hairs, LRX2 has also been implicated in lateral root formation. Similarly, *LRX6* has been found to express during lateral root formation (Baumberger et al., 2003), however, its exact role in root development needs to be investigated. Thus, differentially expressed cell wall- related LRX in chickpea may regulate mechanical stability of cell wall, and support cell expansion during root hair and lateral root development. Peptide hormone, root meristem growth factor 1 (RGF1) is involved in maintaining stem cell niche and root meristem size through ROS signaling in Arabidopsis (Yamada et al., 2020). A RGF gene (LOC101510975) was strongly up-regulated in both chickpea cultivars under K^+^ deficiency, however, another RGF (LOC101507205) showed down-regulation in PUSA362. RADIALIS (LOC113785428, LOC101513794, LOC101491616, LOC101493531, LOC101491616) genes involved in formation of root endodermis (Kaashyap et al., 2018) were found to be down-regulated in both varieties. Overall, the interplay of these important regulators of root development and those which regulate metabolism, signal transduction, phytohormones and TFs could have resulted into variable root growth pattern and RSA in PUSA362 and PUSA372 chickpea cultivars. In future, detail functional investigation and genetic engineering using these crucial genes may help in achieving chickpea plants with desired RSA and improved KUE and K^+^ deficiency tolerance traits.

## 4 Conclusion

In conclusion, among five different chickpea cultivars PUSA362 had shortened primary roots and stunted plant growth under K^+^ deficiency while, PUSA372 had negligible effect on root and overall plant growth. In addition, K^+^ content analysis indicated impaired K^+^ uptake and transportation in PUSA362, but not in PUSA372. These evidences suggest that PUSA362 is a K^+^ deficiency sensitive chickpea cultivar while, PUSA372 is K^+^ deficiency tolerant. Global transcriptome analysis revealed hundreds of K^+^ deficiency responsive genes in PUSA362 and PUSA372. Some crucial genes related to plant metabolism, such as fructose-1,6-bisphosphatase and sucrose synthase were repressed in PUSA362, which could result in low carbohydrate and sucrose level, consequently, hampered transportation of sucrose from shoot (source) to root (sink) and low available energy. Thus, PUSA362 plants may have diverted their limited energy sources in combating K^+^ deficiency than supporting plant growth and resulting in hampered root growth and overall plant growth. An ethylene biosynthesis gene, ACO was up-regulated in PUSA362 while, an ACO gene was down-regulated in PUSA372. As ethylene is known to trigger ROS production and inhibit primary root growth, accumulation of ethylene could be one of the possible reasons for impaired primary root growth in PUSA362 under K^+^ deficiency. Also, an expansin gene which promote root development by controlling cell expansion was down-regulated in PUSA362 but not in PUSA372. In addition to these, interplay of several other hormone related genes (particularly auxin), signal transduction components (like CBLs, CIPKs, PP2C) and TFs (AP2, ARF, MYBs) may determine the chickpea plants response to K^+^ deficiency in two contrasting cultivars. However, adaptive response to stress, such as K^+^ deficiency is a complex trait and may not be completely understood by transcriptional changes. Several other important factors, such as genetic and physiological events, post-translational modifications and epigenetic regulations are potential determinants of an adaptive response. In future, detail functional investigation of some of these key genes and their utilization in genetic engineering and breeding programs will help in understanding the genetic, physiological and molecular basis of K^+^ deficiency tolerance in chickpea. The knowledge so generated will help in developing chickpea plants with better KUE and K^+^ deficiency tolerance traits.

## Data availability statement

The datasets presented in this study can be found in online repositories. The names of the repository/repositories and accession number(s) can be found in the article/**Supplementary Material**.

## Author contributions

AS conceptualized and design the study. AA performed the wet lab experiments. AA, AjS, SK, and AS analyzed the data. AS and AA wrote the manuscript. All authors read and approved the final version of manuscript.
